# Specific Meditation and Specific Medicines

**Published:** 1877-12-20

**Authors:** 


### BOOK NOTICES.

Specific Medication and Specific Medicines. Revised, with an appendix containing the articles published on the subject since the first edition, and a report of cures illustrating Specific Medication. By John M. Scudder, M.D, Professor of the Principles and Practice of Medicine in the Eclectic Medical Institute, etc., etc. Eighth edition. Cincinnati. Wilstach, Baldwin & Co., Printers. 1877.

We had occasion to notice a previous edition of this work. The present edition shows a considerable increase in the size of the volume, and a general improvement over former editions.

Specific Medication is not a new idea under the sun; but Prof. Scudder, albeit he is an Eclectic, gives an entirely different interpretation to that entertained by the profession at large. Contrary to the profession, he believes in specifics. But with the limitation and a proper understanding of his theory, his idea of specifics need not give very great alarm. He, with many others, have found that certain symptoms call for certain remedies. If our patients have pain—say severe neuralgia—we employ morphia, hypodermically. Under these circumstances, the morphia, thus used, would be called by him, we suppose, a specific.

Dr. Scudder employs quite a large list of drugs for their specific effects. We will mention a few, for instance: Has the patient pain located on the abdomen about the umbilicus, he would give nux vomica; for superficial dropsy, wherever located, apocynum canab.; for ministers' sore throat, collinsonia. For hemorrhoids, hamamelis, etc. In fevers, if the pulse is small, he would give aconite; if large, veratrum viride; if the face is flushed and eyes bright, gelseminum. The tongue also affords him indications of specific medication: For the white furred tongue, an alkali is indicated, whereas, when the tongue is red, acids, independent of treatment based upon symptoms—all very good when nothing better points out the way.

The work is of very great value, inasmuch as it treats of remedies of the utmost utility, not often resorted to by the bulk of the profession. And, while no sensible physician will disdain the employment of agents he knows to be of unquestionable value, because employed by Eclectics, he may, if he pleases, object to the theories upon which they may be given, and the doses in which they may be recommended to be administered. Dr. Scudder, we are free to confess, as a representative man of his school, has done the profession, at large, very great service, and his volume, in the hands of the carefully instructed physician, will be helpful; opening up, as it does, a large field from which to draw increased supplies of valuable therapeutic agents.

---

LITTEL'S LIVING AGE.—See prospectus of this excellent magazine in this issue.

